# Rare Differential Diagnosis of Dyspnea: Extramedullary Plasmocytoma (EMP) of the Larynx—Case Report and Review of the Latest Literature of Laryngeal EMP and Laryngeal Involvement of Multiple Myeloma

**DOI:** 10.1155/2019/5654014

**Published:** 2019-04-17

**Authors:** Kim Vanessa Steinke, Barbara Karoline Schneider, Hans Jürgen Welkoborsky

**Affiliations:** ^1^Department of Otorhinolaryngology, Head and Neck Surgery, Nordstadt Clinic, Academic Hospital, Hanover, Germany; ^2^Department of Pathology, Nordstadt Clinic, Academic Hospital, Hanover, Germany

## Abstract

Multiple myeloma (MM) of the larynx is extremely rare. It can be either a laryngeal manifestation of a general multiple myeloma or it can occur as a primary laryngeal mass, which is then called extramedullary plasmocytoma (EMP). We present the case of an 81-year-old male patient who was admitted for dyspnea. He had a history of multiple myeloma but was in complete remission since some years. Histological and immunohistological examination of tissue samples revealed an EMP. The patient was first treated by laser surgery in order to reduce the tumor mass and secure the airway. Afterwards, he was systematically treated by radiation therapy with 60 Gy, which achieved a good response and complete remission proven by control laryngoscopy and histological examination of tissue samples taken from the former tumor area three months after laser excision. The latest literature in the field is reviewed. There were only ten cases of EMP in the larynx or laryngeal involvement of MM published within the last five years (Pubmed was searched for “larynx,” “laryngeal” and “EMP,” “Extramedullary Myeloma,” “Multiple Myeloma,” and “MM”). Due to its rarity, there are currently no evidence-based therapeutic guidelines available. For their development, multicenter studies are required.

## 1. Introduction

Multiple myeloma is a clonal malignant systemic disease of terminal differentiated B cells (plasma cells). The annual incidence in Germany is 4/100000 [1]. It is characterized by accumulation and proliferation of B cells producing monoclonal immunoglobulin. Due to accumulation in the bone marrow, normal hematopoiesis is suppressed and the bone substance is destroyed [1].

These monoclonal plasma cells can also exclusively involve soft tissues, which is then called extramedullary plasmocytoma (EMP). 80–90% of EMP is located in the head and neck which accounts for about 1% of all head and neck tumors [2]. The most affected sites are the oral and nasal cavity, the pharynx, the paranasal sinuses, and the larynx [3]. The main symptoms are due to local invasion of the tumor mass. Men are twice as often affected by EMP than women, whereas the 6th decade of life is the most probable time for disease occurrence [4–7]. Prognostic factors are uncertain [8].

Currently, only few cases of EMP located in the larynx or laryngeal involvement of MM have been reported in the international literature so far. Due to the rarity, to date consistent criteria for staging and treatment do not exist. This makes diagnosis and therapy challenging.

We here present a case of an extramedullary recurrence of a multiple myeloma involving exclusively the larynx.

## 2. Case Report

An 81-year-old male patient was referred to our department with dysphonia. There was no history of smoking. A status past multiple myeloma was known in his medical history that was in complete remission at the time of presentation. The patient denied dyspnea, dysphagia, pharyngalgia, and fever. Laryngoscopy revealed a diminished mobility of the right vocal cord and a thickening of the right vestibular fold so that a microlaryngoscopy with tissue sampling was performed. The histological examination of specimens obtained from this region revealed fibrosis. Computed tomography (CT) scans of the neck and the thorax were without any pathologic findings. The patient was discharged to outpatient care.

**Figure 1:**
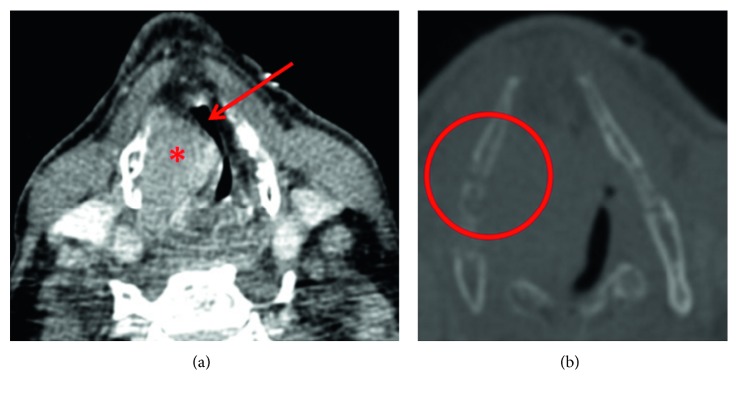
CT scan of the patient with the extramedullary multiple myeloma of the larynx. The tumor displays as a solid mass in the area of the right vocal cord (red asterisk). The lumen of the larynx and therefore also the airway is extremely narrowed (red arrow) (a). The thyroid cartilage on the right side seems to be significantly thinned out (red circle) (b).

Three months later, the patient was admitted with progressive dyspnea along with inspiratory stridor. The clinical examination revealed now a complete paralysis of the right vocal cord and a remaining glottic cleft of only 1 mm due to a supraglottic protrusion of the right vestibular fold. The CT scan (Figure 1) showed now a tumor of the right vocal cord extending to the right piriform sinus.

**Figure 2:**
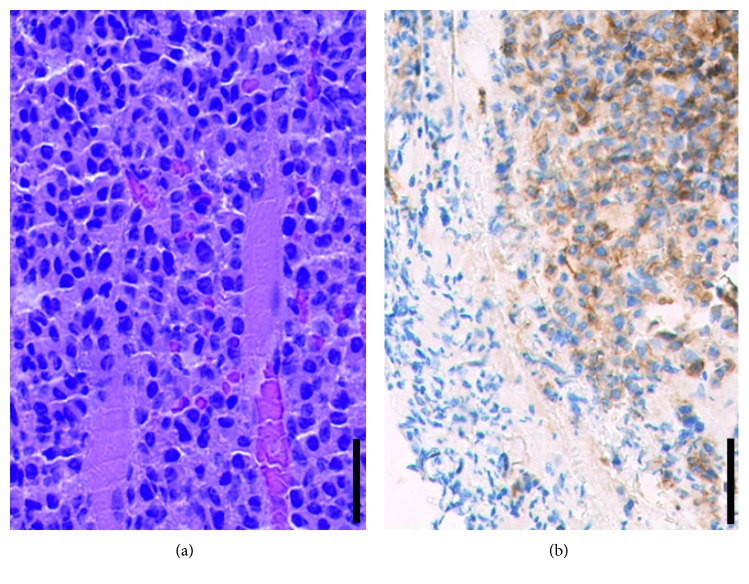
In HE staining, tight lymphoid infiltrates can be seen. The cells have large nuclei, and they are irregularly constructed (a). Immunohistochemical staining reveals a positive expression of CD10 (b). Magnification: 40x; scale bar: 50 *μ*m.

After tumor debulking in order to expand and secure the airway, the excised material that consisted of several red brown elastic tissue fragments measuring together 24 × 12 × 10 mm was sent for pathological examination. Histologically, one could see tight lymphoid infiltrates. The cells had large nuclei and were irregularly shaped, and the proliferation was strongly enhanced in the staining for Ki67 (50%). The immunohistochemical analyses showed a negative result for CD20 and CD3, and a positive staining for CD138. BCL2 and CD10 were coexpressed (Figure 2). The clonal light chain restriction for lambda chains substantiated the diagnosis of a multiple myeloma. These results were consistent with laryngeal involvement from the patient's previously diagnosed multiple myeloma.

The patient was referred to the Department of Hematology and a systemic therapy with the proteasome inhibitor Bortezomib was discussed. Ultimately, instead of that, a local radiation therapy with 60 Gy was performed. In a control laryngoscopy with tissue sample taken after the radiotherapy, the myeloma could not be verified anymore. The patient is in continuous otorhinolaryngological and oncologic follow-up. To date, almost two years later, no recurrence of the myeloma has occurred so far.

## 3. Discussion

The monoclonal terminally differentiated plasma cells of MM occur in the bone marrow as well as in soft tissues. Usually, patients with MM suffer from weakness, loss of weight, an increased susceptibility for infections, osteoporosis, pathologic fractures, hyperviscosity syndrome, or nephrotic syndrome [4–7]. The median age at diagnosis is 50–80 years [9], and men are slightly predilected [10, 11]. When only the extramedullary soft tissues are affected, the disease is called EMP. In 80% of cases, EMP involves the upper aerodigestive tract [11].

The laryngeal involvement is extremely rare and then can occur primarily as EMP (6–18%) [12] or secondarily after medullary MM. The laryngeal infiltration of MM or EMP accounts for 0.04–0.19% of all laryngeal tumors [13]. Anatomical laryngeal sites which are mainly affected are the epiglottis, vocal cords, false vocal folds, aryepiglottic folds, and thyroid cartilage [3]. Main symptoms appear due to the local involvement and may include hoarseness, dyspnea, dysphagia, hemoptysis, and stridorous breathing. As EMP arises from the subepithelial layer, it is essential to take deep submucosal samples for histological examination in order to verify the diagnosis [3].

The histological examination is challenging because a clear delineation to other pathologies, i.e., chronic inflammatoric diseases or plasma cell enriched polyps as well as amyloid deposit, can be difficult [14, 15]. Therefore, immunohistochemistry, flow cytometry, and immunophenotyping are crucial. Flow cytometry helps to characterize the cell population of the specimen by determining the cell size and the heterogeneity of the cells. Furthermore, by immunohistochemistry and fluorescence-activated cell sorting (FACS), it is possible to detect the expression of certain cell surface antigens (i.e., CD 38 and CD 138). These results help to prove the diagnosis of EMP [16].

Imaging techniques, i.e., computed tomography or magnetic resonance tomography, are helpful in order to clarify the local involvement of anatomical structures as well as to exclude further osseous and soft-tissue lesions and lymphadenopathy [17]. Typical findings for EMP in computed tomography imaging are calcification and areas with low densities in the thyroid cartilage and infiltrative growth pattern [18, 19].

**Figure 3:**
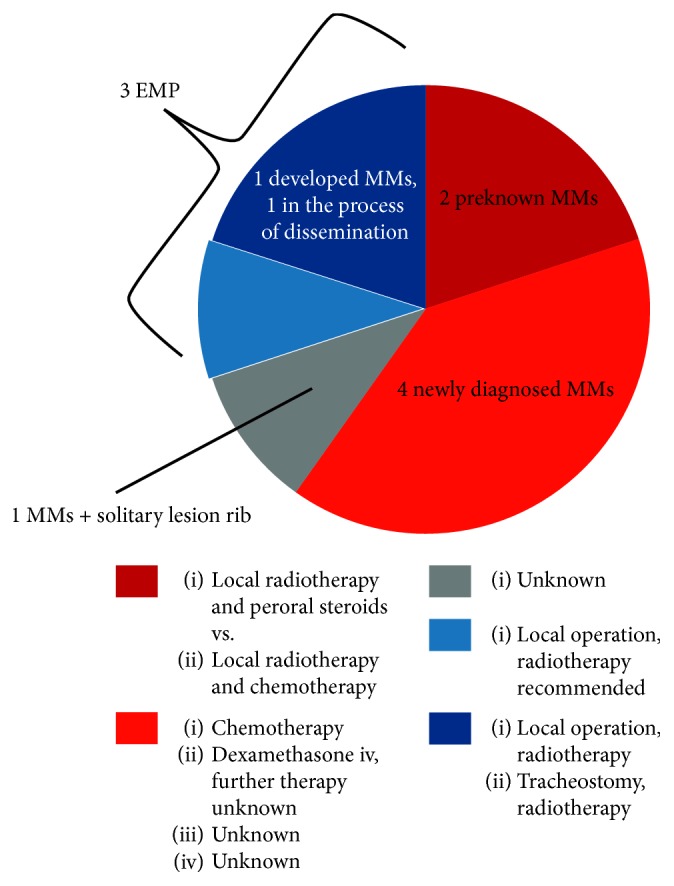
Pie chart of the distribution of MM and EMP and chosen therapy in the cases published in the literature during the past five years concerning laryngeal involvement by plasmocytoma (*n* = 10).

Here, we have reviewed the latest literature. Ten cases of MM in the larynx or laryngeal EMP have been published in the last five years according to our Pubmed search [3, 8, 20–27]. The clinical and therapeutical findings are detailed in Table S1. In two of the ten cases, a MM had been known in the patient's history [3, 22]. In four of the remaining eight cases, the pathologic clinical findings in the larynx led to the diagnosis of MM [20, 21, 25, 26]. In one of the remaining four cases, additionally to the findings in the larynx, there was a solitary plasmacytoma of the rib [24]. There is no information provided concerning the follow-up of this patient. The three remaining cases can be regarded as solitary EMP [8, 23, 27]. In one of these three cases, the patient developed a MM three years and four months after diagnosis of EMP, which can be regarded as a progression from EMP to MM [27]. In one of the other cases, the diagnosis of isolated primary EMP in the process of dissemination was most appropriate [23] (Figure 3).

As far as therapy is concerned, it is the experience of the published cases in the last five years that the patients with preknown MM benefit from local treatment with radiation therapy and subsequent systemic therapy [3, 22]. Patients with newly diagnosed MM were systemically treated [20, 21], whereas in patients with EMP, surgery for local excision of tumor masses followed by radiotherapy was recommended [8, 23, 27].

The treatment performed in the present case fits well in the series of similar cases in the literature.

The cases of the last five years show that there is no overall consensus regarding therapy for laryngeal EMP due to its rarity. There is some evidence that it is well treatable with radiotherapy with a good local control [23]. Alternatively local operative therapy, i.e., with CO_2_-laser excision in combination with radiotherapy, might be appropriate [8, 27]. The decision for the appropriate individual therapy should always be made in a multidisciplinary setting.

Autologous or allogenic stem cell transplantation is regarded as further alternatives to systemic therapy of MM [28]. As there is to date no cure of MM, a life-long oncologic follow-up is essential [29]. Reviewing the literature papers published on this issue possessed several limitations. Most of the papers contain case reports and no cohort studies. Furthermore, no uniformity could be found in the publications regarding the stage, grade, and outcomes, which makes a comparability of these reports difficult. Currently, no evidence-based therapy standards are available for extramedullary plasmocytoma [20]. Therefore, multicenter studies should be suitable and recommended to develop therapy guidelines.

## 4. Conclusion

In conclusion, EMP of the larynx or laryngeal involvement of MM is extremely rare. It should be taken under consideration in cases with subepithelial diseases of the larynx. For histological examination, a deep and submucosal tissue sample is mandatory and diagnosis can only be made histologically and immunohistologically since urinary Bence Jones proteins or serum multiple myeloma protein can only be found occasionally [27].

## Supplementary Materials

Supplementary Materials Table 1: overview of the cases of MM or EMP of the larynx published during the last five years [3, 8, 20–27]. The table shows age, sex, symptoms, anatomical site, treatment, response to therapy, and follow-up of the cases, if known. Besides, it is listed whether MM was preknown in the medical history of the patient.
