# Supplementary material for: Rare Differential Diagnosis of Dyspnea: Extramedullary Plasmocytoma (EMP) of the Larynx—Case Report and Review of the Latest Literature of Laryngeal EMP and Laryngeal Involvement of Multiple Myeloma
Source: Case Rep Otolaryngol. 2019 Apr 17;2019:5654014. doi: 10.1155/2019/5654014 (PMC6501167; doi:10.1155/2019/5654014)
Supplement: Supplementary Materials — Table 1: overview of the cases of MM or EMP of the larynx published during the last five years [3, 8, 20–27]. The table shows age, sex, symptoms, anatomical site, treatment, response to therapy, and follow-up of the cases, if known. Besides, it is listed whether MM was preknown in the medical history of the patient. [file 5654014.f1.pdf]

	Alherabi (20)	Allegra (21)	Flore (22)	Ghatak (23)	Haser (24)	Mitchell (3)	Nochikattil (25)	Oral (26)	Wang (27)	Xing (8)
**Age**	77	68		29		63	44	43	43	47
**Sex**	M	M	M	F	?	M	F	M	M	F
**Symptoms**	dysphagia	dyspnea, dysphagia, dysphonia	dyspnea	hoarseness, dysphagia, intermittent respiratory difficulty	?	hoarseness, neck mass	hoarseness, stridor	pain of the left leg, no symptoms of the larynx	hoarseness, dyspnea	hoarseness
**Anatomical Site**	diffuse supraglottic swelling with ill-defined epiglottic and hypopharyng-eal mass	lesion involving the right glottis and right vestibular (false) vocal fold, constriction of the airway	arising from the cricoid, thinning and expansion of the cartilage laminae without mucosal lesions	fleshy mass involving the posterior third of the true vocal cords, encroaching on the ventricle and false cords	cricoid cartilage with solitary plasmacytoma of the rib	bulging left false vocal fold under normal mucosa	mucosa covered smooth swelling over left arytenoid, aryepiglottic fold and left piriform sinus	thyroid cartilage and bones	swelling of the arytenoid, vocal cord, laryngeal ventricle and ventricular band	2x2cm reddish smooth mass overlying the left hemilarynx, the base from the aryepiglottic fold
**pre-known MM?**	no	no	yes	no	no	yes	no	no	no	no
**Treatment**	dexamethasone iv and nasogastric tube feeding, further therapy alio loco	6 cycles of chemotherapy (bortezomib, thalidomide + dexamethaso-ne)	radiation therapy + peroral steroids	tracheostomy, radiotherapy	?	chemotherapy with lenalidomide + external beam radiotherapy to the larynx	referred to oncology	?	tracheostomy, complete excision of the invaded tissue, radiotherapy refused	tracheostomy, complete excision via lateral neck incision, postoperative radiotherapy
**Response**	good response	partial remission	good response	good response	?	good response	?	?	tumor recurrence after 1 year → operation, 2nd recurrence with osseous masses → chemotherapy with melphalane, prednisone + thalidomide	neck abscess 6 months after therapy, no recurrence
**Follow-up**	no	yes, interferone and steroid	?	yes	?	yes	?	?	yes	yes
